# Effects of secukinumab and ixekizumab on major adverse cardiovascular events in patients with psoriasis: a meta-analysis of randomized controlled trials

**DOI:** 10.3389/fmed.2024.1353893

**Published:** 2024-03-06

**Authors:** Yonghong Zhang, Zhiya Yang, Jinyan Gong, Dongmei Shi

**Affiliations:** ^1^Shandong University of Traditional Chinese Medicine, Jinan, China; ^2^Laboratory of Medical Mycology, Jining No. 1 People’s Hospital, Jining, China; ^3^Department of Dermatology, Jining No. 1 People’s Hospital, Jining, China

**Keywords:** secukinumab, ixekizumab, psoriasis, major adverse cardiovascular events, adverse event

## Abstract

**Introduction:**

The aims of this study is to analyze the risk of major adverse cardiovascular events (MACEs) in patients with psoriasis treated with secukinumab and ixekizumab.

**Methodology:**

We systematically identified randomized controlled trials (RCTs) that focused on the treatment of psoriasis with secukinumab and ixekizumab by conducting computerized searches of PubMed, Embase, and the Cochrane Library databases, spanning from their inception to October 31st, 2022. The search terms used included psoriasis, secukinumab, ixekizumab, and randomized controlled trial. Two independent evaluators conducted literature screening, data extraction, and assessed the quality of included studies based on predetermined inclusion and exclusion criteria. The gather data was subjected to meta-analysis using the statistical software RevMan 5.4.

**Results:**

A total of 20 articles, encompassing 23 randomized controlled trials involving 10,746 psoriasis patients were included in the analysis. During the double-blind treatment period, the meta-analysis results indicated the following: There was no significant difference in the incidence of MACEs between the secukinumab and placebo groups [RR = 0.61, 95% CI (0.26, 1.44), *p* = 0.26]. Similarly, there was no significant difference in the incidence of MACEs with ixekizumab compared to the placebo group [RR = 0.47, 95% CI (0.15, 1.47), *p* = 0.20]. Furthermore, no significant difference in the incidence of MACEs was observed between secukinumab 300 mg and secukinumab 150 mg treatment groups [RR = 1.00, 95% CI (0.23, 4.35), *p* = 1.00]. Likewise, there was no significant difference in the incidence of MACEs between the ixekizumab Q4W (every 4 weeks) and ixekizumab Q2W (every 2 weeks) administration groups [RR = 4.01, 95% CI (0.45, 35.89), *p* = 0.21].

**Conclusion:**

The findings of this study suggest that neither secukinumab nor ixekizumab is significantly associated with the risk of MACEs in patients with psoriasis during double-blind treatment.

**Systematic review registration**: Unique Identifier: CRD42022373756 https://www.crd.york.ac.uk/.

## 1 Introduction

Psoriasis is a systemic inflammatory disease with diverse clinical manifestations and comorbidities that significantly impact the quality of life for affected individuals (1). Unlike the general population, individuals with psoriasis experience effects on almost every system in the body. Among the various conditions associated with psoriasis, cardiovascular (CV) disease stands out as a major concern due to its common occurrence and immediate implications for morbidity and mortality (2). The connection between cardiovascular disease, as one of these comorbidities, and medications used in the treatment of psoriasis has become a subject of discussion as attention on psoriasis-related comorbidities increase. Research indicates a consistent association between severe psoriasis and an elevated risk of Major Adverse Cardiovascular Events (MACEs), with medications employed in psoriasis treatment also exhibiting a connection to the development of cardiovascular disease (3, 4).

Pooled data analysis reveals that biologic treatments contribute to a reduction in CV events, coronary inflammation, and the presence of high-risk coronary plaques. In particular, anti-interleukin (IL)-17 therapy has shown positive effects on systemic inflammation and coronary plaque in patients with psoriasis (5). The introduction of biologics, specifically those inhibiting IL-17, has provided new options for the maintenance therapy of various autoimmune diseases. Currently approved in China, IL-17A inhibitors such as secukinumab and ixekizumab are human monoclonal antibodies that directly targeting interleukin-17A (IL-17A) (6). Studies conducted on the Chinese population have demonstrated that subcutaneous injections of secukinumab and ixekizumab are highly effective, provide rapid relief of symptoms, and have fewer adverse reactions (7, 8).

Hence, the purpose of this study is to systematically review the published randomized controlled trials involving secukinumab and ixekizumab in the treatment of psoriasis. The objective is to evaluate the risk of MACEs and provide recommendations for cautions clinical utilization.

## 2 Materials and methods

### 2.1 Study design and selection

**Figure 1:**
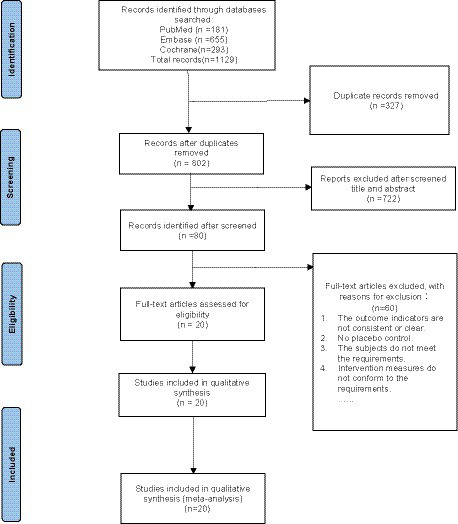
PRISMA flow diagram.

A comprehensive literature search method was utilized to systemic examine all relevant literature from the inception of PubMed, Embase, and Cochrane Library databases up to October 2022, without any language restrictions. This study included randomized controlled trials (RCTs) involving secukinumab and ixekizumab in the treatment of psoriasis. The search employed specific terms such as “secukinumab,” “ixekizumab,” “psoriasis,” “randomized controlled trial,” and others, combining subject headings and free-text terms. A detailed overview of the retrieval strategy is presented in Figure 1.

### 2.2 Population of interest

The study specifically focused on adult patients aged 18 years or older who had been diagnosed with psoriasis for a duration of at least 6 months, regardless of gender. Psoriasis diagnoses were confirmed by dermatologists through examination and biopsy, while diagnoses of psoriatic arthritis (PsA) were made by rheumatologists following the CASPAR diagnostic criteria.

Exclusion criteria included patients below the age of 18, individuals unwilling to discontinue their current treatment regimen, participation in other clinical trials within the last 3 months, non-randomized controlled studies, reviews, animal studies, conference abstracts, and cases where the full text was inaccessible or data extraction was incomplete.

### 2.3 Interventions and comparators

In the experimental group, subcutaneous administrations of secukinumab or ixekizumab were given. The dosage of secukinumab was set at 300 mg and 150 mg, respectively, while the dosage of ixekizumab varied in frequency, either once every 2 weeks or once every 4 weeks. The control group received subcutaneous placebos, encompassing both no treatment and treatment-as-usual.

### 2.4 Outcomes measure

This meta-analysis aimed to examine the impact of IL-17A inhibitors on MACEs in patients with psoriasis. The analysis focused on four key aspects:

- The incidence of MACEs in patients with psoriasis who treated with secukinumab;
- The evaluation of the incidence of MACEs with secukinumab at 300 mg and 150 mg;
- The assessment of MACEs incidence in patients with psoriasis treated with ixekizumab;
- Comparative analysis of the incidence of MACEs with different frequencies of ixekizumab administration.

### 2.5 Data extraction

Using the Endnote X9 literature management software, two researchers conducted a thorough screening of the literature, strictly adhering to pre-established inclusion and exclusion criteria. The initial step involved eliminating duplicate literature, followed by a detailed review of the article’s title and abstract for preliminary screening. Upon obtaining the full text, a secondary filtration process was carried out. Any discrepancies encountered during the screening process were resolved through negotiation or consultation with a third party. The extracted data were meticulously recorded in tabular form, capturing key details such as the first author, year of publication, country of publication, experimental design, basic characteristics of study subjects, sample size, intervention measures, outcome indicators, duration of disease, and the incidence of MACEs during the trial.

### 2.6 Assessment of risk of bias for included studies

The methodological rigor of the incorporated studies was assessed utilizing the bias risk assessment tool designed for randomized controlled trials, as stipulated in the Cochrane Manual of Systematic Reviewers 5.1.0.

### 2.7 Statistical analysis

The comparison of MACEs between secukinumab and ixekizumab, as well as their comparison with a placebo was conducted using RevMan 5.4 software. Relative risk served as the effect statistic for dichotomous variables was expressed as the relative risk (RR), and a 95% confidence interval (CI) was employed for interval estimation. Statistical heterogeneity was assessed using the chi-square test, and the extent of heterogeneity was measured using *I*^2^ test. If no significant heterogeneity was observed (*p* > 0.10, *I*^2^ ≤ 50%), the fixed-effects model was applied. In case of where heterogeneity was present, a thorough analysis of its potential causes was conducted, and subsequently, the random-effects model was implemented.

## 3 Results

### 3.1 Study characteristics

Through a systematic search, a total of 1,129 articles were retrieved, distributed across PubMed (*n* = 181), Embase (*n* = 655), and the Cochrane Library (*n* = 293). After initial screening, 81 articles were selected for further evaluation. Following a rigorous secondary screening process, 20 articles were included for both qualitative and quantitative analysis. It worth noting that all of included articles were in English (Figure 1).

### 3.2 Risk of bias

In conclusion, the analysis included a total of 20 studies, encompassing 23 randomized controlled trials, which involved 10,746 participants. The experimental group consisted of 7,738 participants, while the control group included 3,008 participants. The essential information from these studies is presented in Table 1.

**Table 1:** Characteristics of the included studies.

First Author, surname, and Year	Country	Design	Group	Sample size	Male	MACEs	Age, y	Duration of psoriasis, y	treatment period, w
Bagel, J., 2017	United States	RCT	(1) Secukinumab 300 mg treatment group	51	27	0	42.7 ± 13.4	NA	12
			(2) Placebo control group	51	24	0	41.1 ± 14.2		
Nash, P., 2018	Australia	RCT	(1) Secukinumab 300 mg treatment group	139	67	0	49.3 ± 12.9	NA	16
			(2) Secukinumab 150 mg treatment group	138	61	0	50.1 ± 11.7		
			(3) Placebo control group	137	59	0	50.1 ± 12.6		
Papp, K., 2013	Canada	RCT	(1) Secukinumab 450 mg treatment group	27	21	0	45.4 ± 11.64	16.2 ± 10.41	12
			(2) Secukinumab 225 mg treatment group	21	14	1	45.8 ± 12.36	17.1 ± 9.01	
			(3) Secukinumab 75 mg treatment group	26	22	3	46.3 ± 13.43	17.7 ± 11.53	
			(4) Secukinumab 25 mg treatment group	29	20	1	46.1 ± 12.65	19.8 ± 12.66	
			(5) Placebo control group	22	14	3	45.9 ± 10.88	21.4 ± 14.80	
Paul, C., 2015	France	RCT	(1) Secukinumab 300 mg treatment group	60	46	0	46.6 ± 14.23	21.0 ± 13.51	12
			(2) Secukinumab 150 mg treatment group	61	41	0	43.9 ± 14.41	20.6 ± 14.54	
			(3) Placebo control group	61	38	0	43.7 ± 12.74	19.86 ± 12.20	
Blauvelt, A., 2015	United States	RCT	(1) Secukinumab 300 mg treatment group	59	38	2	45.1 ± 12.57	18.0 ± 11.86	12
			(2) Secukinumab 150 mg treatment group	59	40	0	46.0 ± 15.09	20.4 ± 12.97	
			(3) Placebo control group	59	39	0	46.5 ± 14.14	20.2 ± 14.22	
Ohtsuki, M., 2014	Japan	RCT	(1) Secukinumab 300 mg treatment group	29	26	0	51.9 ± 11.77	15.6 ± 10.30	12
			(2) Secukinumab 150 mg treatment group	29	23	1	48.2 ± 13.08	15.6 ± 10.41	
			(3) Placebo control group	29	23	0	50.2 ± 13.62	14.1 ± 10.91	
Mrowietz, U., 2019	United States	RCT	(1) Secukinumab 300 mg treatment group	79	15	0	50.6 ± 14.8	NA	16
			(2) Secukinumab 150 mg treatment group	80	17	0	50.7 ± 13.7		
			(3) Placebo control group	78	19	1	52.9 ± 11.3		
Langley, R., 2014	United Kingdom	RCT	(1) Secukinumab 300 mg treatment group	245	169	0	44.9 ± 13.5	17.4 ± 11.1	12
			(2) Secukinumab 150 mg treatment group	245	168	0	44.9 ± 13.3	17.5 ± 12.0	
			(3) Placebo control group	248	172	0	45.4 ± 12.6	17.3 ± 12.4	
Nguyen, T., 2022	California	RCT	(1) Secukinumab 300 mg treatment group	103	53	0	51.9 ± 12.6	NA	16
			(2) Secukinumab 150 mg treatment group	103	56	1	51.3 ± 14.6		
			(3) Placebo control group	52	23	1	53.1 ± 12.7		
Mease, P. J., 2015	United Kingdom	RCT	(1) Secukinumab 150 mg treatment group	202	96	0	49.6 ± 11.8	NA	16
			(2) Secukinumab 75 mg treatment group	202	84	1	48.8 ± 12.2		
			(3) Placebo control group	202	96	0	48.5 ± 11.2		
Gottlieb, A., 2017	United States	RCT	(1) Secukinumab 300 mg treatment group	69	38	0	48.8 ± 14.2	NA	16
			(2) Secukinumab 150 mg treatment group	68	40	0	52.4 ± 12.6		
			(3) Placebo control group	68	34	0	50.9 ± 13.0		
McInnes, I. B., 2015	United Kingdom	RCT	(1) Secukinumab 300 mg treatment group	100	51	0	46.9 ± 12.6	NA	16
			(2) Secukinumab 150 mg treatment group	100	55	0	46.5 ± 11.7		
			(3) Secukinumab 75 mg treatment group	99	47	1	48.6 ± 11.4		
			(4) Placebo control group	98	39	0	49.9 ± 12.5		
Reich, K., 2019	Germany	RCT	(1) Secukinumab 300 mg treatment group	66	53	0	45.1 ± 12.9	18.01	16
			(2) Secukinumab 150 mg treatment group	67	55	0	43.5 ± 10.9	20.02	
			(3) Placebo control group	65	52	0	43.6 ± 11.2	17.35	
Kemény, L., 2019	Hungary	RCT	(1) Ixekizumab treatment group	734	NA	3	45.4 ± 13.1	18.4 ± 12.3	12
			(2) Placebo control group	360		1			
Ryan, C., 2018	Ireland	RCT	(1) Ixekizumab treatment group	75	56	0	43.1 ± 13.0	16.9 ± 12.8	12
			(2) Placebo control group	74	57	0	44.4 ± 12.6	16.1 ± 12.5	
Nash, P., 2017	Australia	RCT	(1) Ixekizumab treatment group (Q4W)	122	59	0	52.6 ± 13.6	15·7 ± 12.3	24
			(2) Ixekizumab treatment group (Q2W)	123	73	0	51·7 ± 11.9	16.5 ± 13.0	
			(3) Placebo control group	118	62	2	51·5 ± 10.4	15.3 ± 12.6	
Mease, P. J., 2017	United States	RCT	(1) Ixekizumab treatment group (Q4W)	107	45	0	49.1 ± 10.1	16.5 ± 13.8	24
			(2) Ixekizumab treatment group (Q2W)	103	48	0	49.8 ± 12.6	17.0 ± 14.0	
			(3) Placebo control group	106	48	0	50.6 ± 12.3	16.0 ± 13.8	
Gordon, K. B., 2016	United States	RCT	(1) Ixekizumab treatment group (Q4W)	1,165	791	2	*	*	12
			(2) Ixekizumab treatment group (Q2W)	1,169	766	0			
			(3) Placebo control group	792	560	1			
Griffiths, C. E. M., 2015	United Kingdom	RCT	(1) Ixekizumab treatment group (Q4W)	733	502	1	*	*	12
			(2) Ixekizumab treatment group (Q2W)	736	475	0			
			(3) Placebo control group	361	257	1			
Leonardi, C., 2012	United States	RCT	(1) Ixekizumab treatment group	115	67	0	*	*	20
			(2) Placebo control group	27	27	0			

*Details are provided in the Supplementary Table S1; NA, Not mentioned.

**Figure 2:**
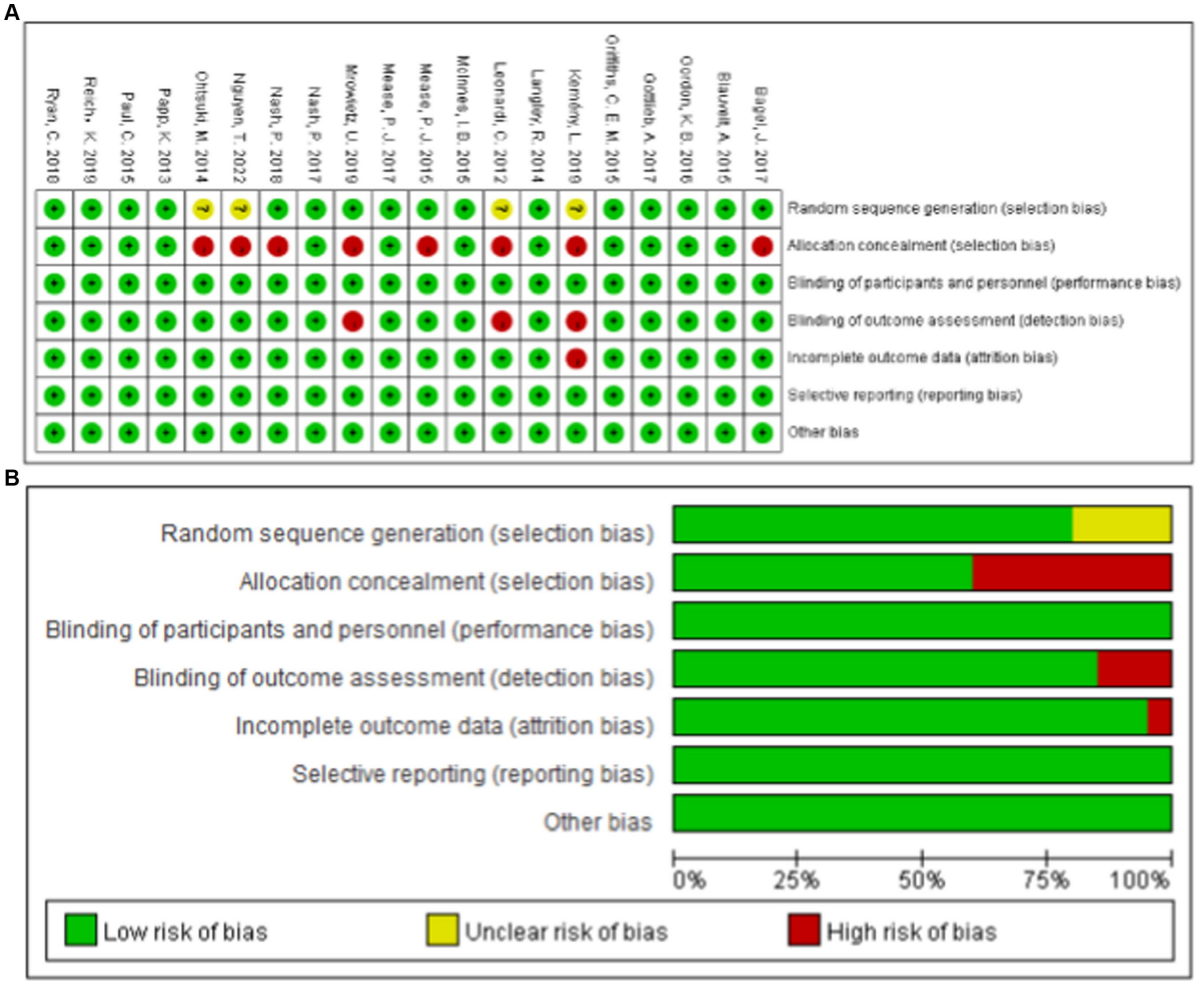
Summary of Cochrane risk of Bias for the RCTs.

All of the studies adhered to a randomized double-blind trial design. However, four studies (9–12) exhibited had unclear randomization methods, one study (11) lacked clarity in outcome index evaluation methods, and 12 studies (13–24) utilized distributive hiding. Apart from one study (20), the remaining 19 studies were conducted at multiple centers. It should be noted that all studies experienced participant due to adverse reactions, such as psoriatic erythroderma, alopecia, herpes and others. A detailed breakdown of the bias risk assessment results is illustrated in Figure 2.

### 3.3 Meta-analysis

#### 3.3.1 Incidence of MACEs in patients with psoriasis using secukinumab

**Figure 3:**
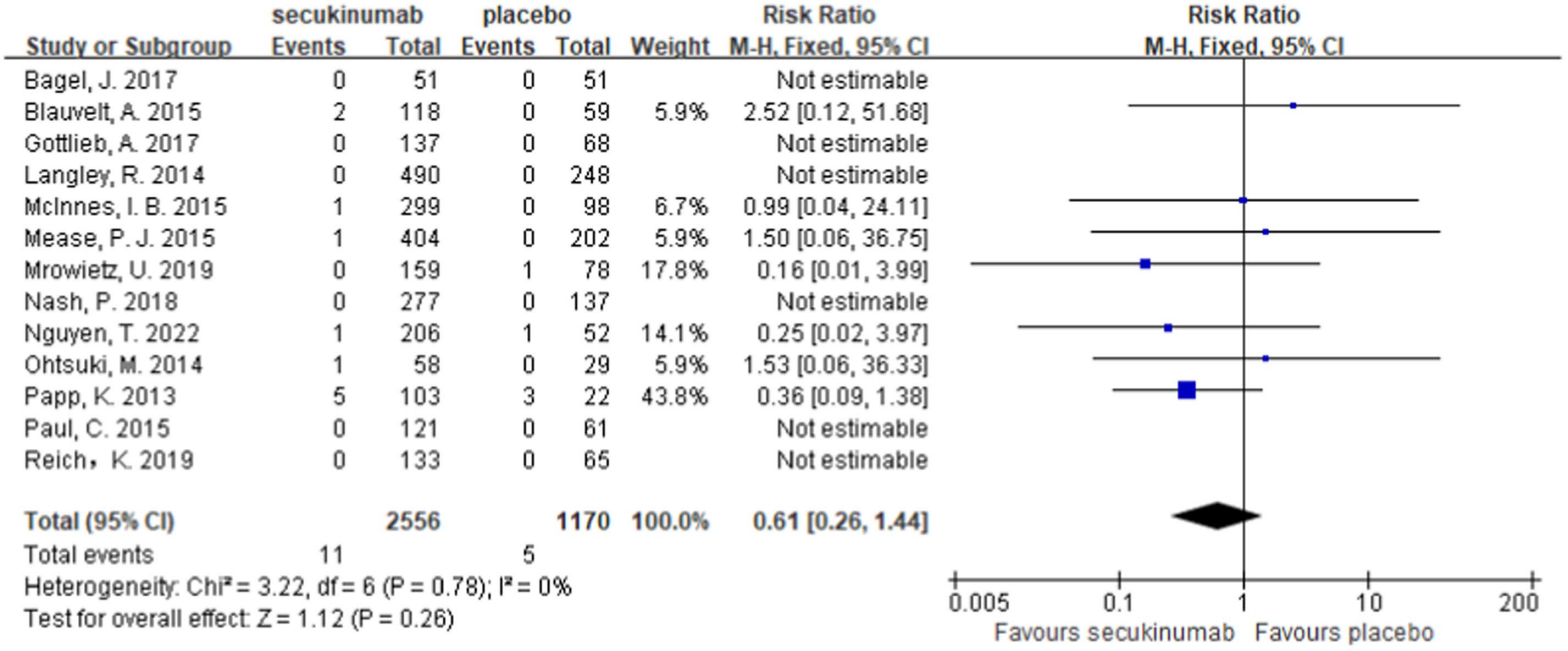
Forest plots of the incidence of MACEs between secukinumab and placebo groups.

Thirteen studies reported MACEs in patients treated with secukinumab (9, 10, 13–19, 25–28). The results of meta-analysis showed that there was no increase in the incidence of MACEs in patients treated with secukinumab. Furthermore, there was no significant difference between the experimental group and the control group [RR = 0.61, 95%CI (0.26, 1.44), *p* = 0.26] as depicted in Figure 3.

#### 3.3.2 Comparison of secukinumab in the incidence of 300 mg and 150 mg MACEs

**Figure 4:**
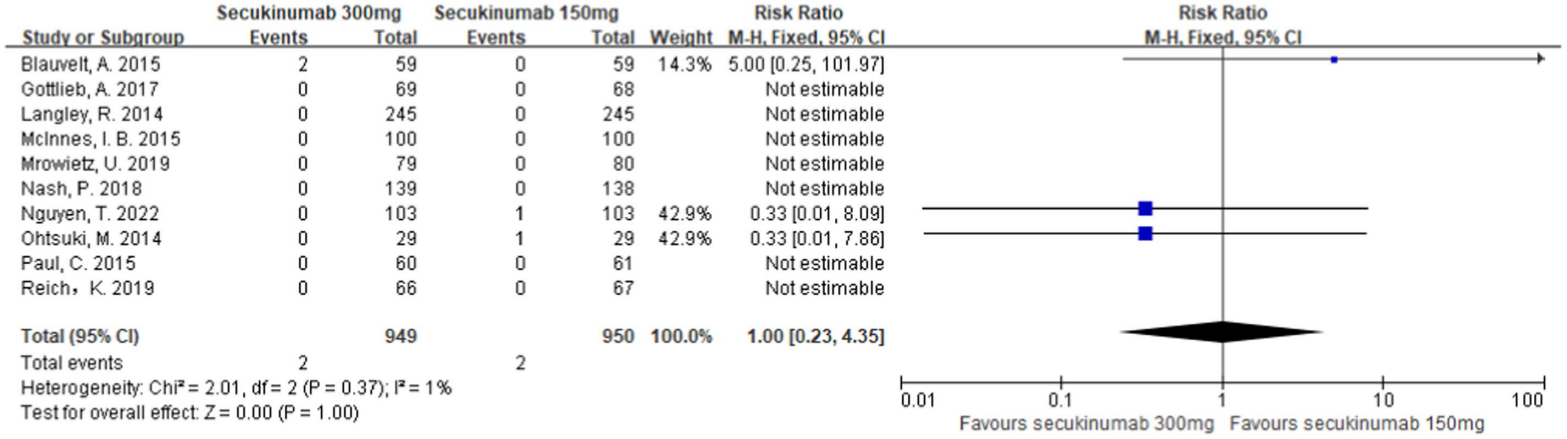
Forest plot of the incidence of MACEs between the 300 mg and 150 mg secukinumab groups.

Ten studies (9, 10, 14–19, 26, 27) have reported a direct comparison of the incidence of MACEs in patients treated with secukinumab at different doses of 300 mg and 150 mg. The meta-analysis results showed that there was no significant difference between the two groups [RR = 1.00, 95%CI (0.23, 4.35) *p* = 1.00] as shown in Figure 4.

#### 3.3.3 Incidence of MACEs in patients with psoriasis using ixekizumab

**Figure 5:**
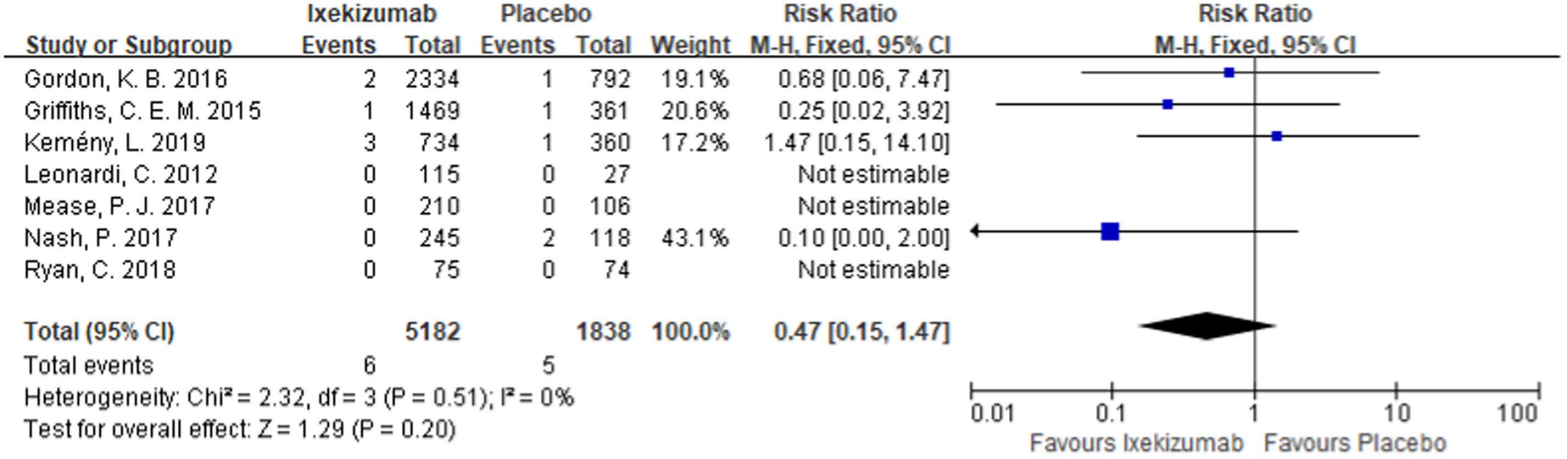
Forest plot of incidence of MACEs between ixekizumab and placebo groups.

The incidence of MACEs in patients with psoriasis treated with ixekizumab was reported in seven studies (11, 12, 20–24). The meta-analysis results showed that ixekizumab did not increase the incidence of MACEs in patients. Additionally, there was no significant difference between the experimental group and the control group [RR = 0.47, 95%CI (0.15, 1.47), *p* = 0.20] as depicted in Figure 5.

#### 3.3.4 Comparison of the incidence of different frequencies of administration of ixekizumab

**Figure 6:**
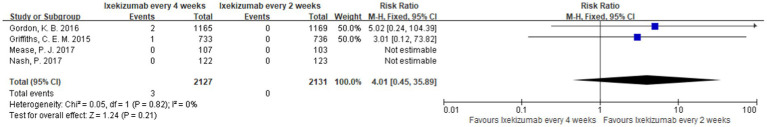
Forest map of the incidence of MACEs between the different frequencies of administration of ixekizumab.

A direct comparison of the incidence of MACEs in patients treated with different frequencies of administration of ixekizumab (receive subcutaneous injections of ixekizumab every 2 weeks and every 4 weeks) was reported in four studies (21–24). The meta-analysis results showed that there was no significant difference between the two groups [RR = 4.01, 95%CI (0.45, 35.89) *p* = 0.21] as shown in Figure 6.

### 3.4 Bias in publication

**Figure 7:**
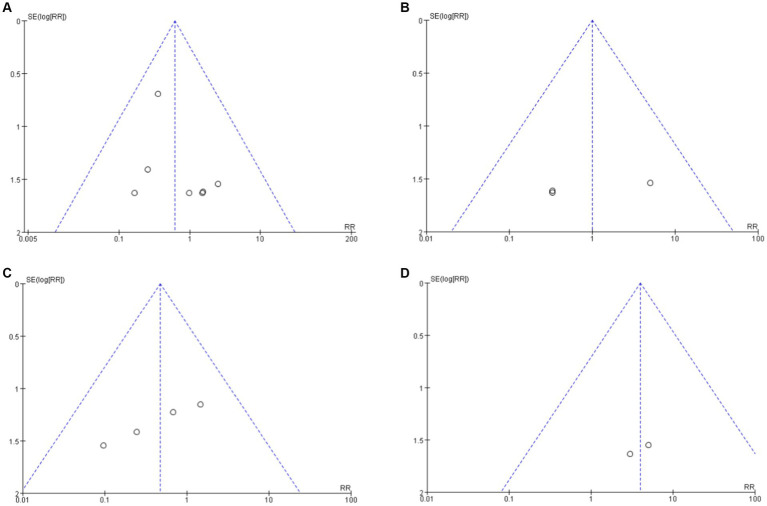
Funnel plot analysis of clinical indicators among each group. **(A)** Secukinumab vs. Placebo. **(B)** Secukinumab 300 mg vs. 150 mg. **(C)** Ixekuzumab vs. Placebo. **(D)** Ixekizumab Every 4 Weeks vs. Every 2 Weeks.

Funnel plots were used to analyze publication bias in the literatures reporting the incidence of MACEs in each group (Figure 7). The results showed that all funnel plots had good symmetry, suggesting that the study results were less affected by publication bias.

## 4 Discussion

### 4.1 Quality of the studies

The search strategy employed is robust and designed to minimize the likelihood of overlooking eligible studies. Most of the included studies exhibit a clear and high risk of bias (ROB), with explicit references to randomization and blinding strategies. For example, Nash et al. (21) implemented treatment allocation concealment and generated a random sequence using a computer. The study ensured that investigators, study site staff, and patients remained unaware of treatment allocations until the initial data analysis. However, a few studies lack clarity in their descriptions of randomization and blinding methods. In the study conducted by Nguyen et al. (10), for instance, the randomization method was unclear, and no specific treatment allocation scheme was specified.

### 4.2 Outcome

Psoriasis, a chronic inflammatory skin disease, has a significant impact on both the physical and mental well-being of individuals affected by it. Emerging evidence suggests that severe psoriasis is not only linked to metabolic disorders, obesity, and heightened mortality but also serve as an independent risk factor for atherosclerosis, myocardial infarction, and stroke (29). The condition is associated with various comorbidities, including cardiovascular disease, metabolic syndrome, cancer, gastrointestinal disorders, and chronic obstructive pulmonary disease (COPD) (30). A recent study conducted in UK revealed that individuals with moderate to severe psoriasis had a lifespan that was approximately 6 years shorter than their healthy counterparts, possibly attributed to inflammation-induced cardiovascular diseases (CVD), such as myocardial infarction and cerebral infarction (31).

Furthermore, research indicates a 57% increased risk of cardiovascular (CV) death among patients with severe psoriasis compared to those with traditional CV risk factors. Notably, while the majority of CV death risks are typically attributed to explained by major cardiac risk factors, this association is not consistently observed in severe psoriasis cases, implying an independent association between severe psoriasis and CV death (32). Additionally, Psoriatic Arthritis (PsA) is linked to a higher incidence of CVD complications in Western countries and Japan. Recent analyses of cytokines from psoriasis patients suggest that certain cytokines, including tumor necrosis factor (TNF)-α, interleukin (IL)-17, and IL-23, have cardiovascular effects (31).

The IL-17A inhibitors such as secukinumab and ixekizumab have been approve for the treatment of psoriasis in China. Many studies have shown that the efficacy and safety of these two drugs in treating psoriasis, but their impact on psoriasis comorbidities, particularly cardiovascular disease has received less attention. Therefore, studies have been conducted to investigated the effect of IL-17A inhibitors on cardiovascular events in patients with psoriasis.

This study included 20 studies to investigate the association between IL-17A inhibitors and psoriasis comorbidities (specifically cardiovascular disease) based on the occurrence of MACEs in patients with psoriasis treating with secukinumab and ixekizumab. With the exception of one study, the other included studies were multi-center randomized double-blind controlled trials with well-defined inclusion and exclusion criteria. Among the 20 studies, only 4 studies had a large bias due to unclear randomization methods, while most of the studies had a low risk of bias. The meta-analysis results of this study showed no significant difference in the incidence of MACEs between secukinumab and ixekizumab compared to placebo, and the difference between the two groups was not statistically significant. The findings were consistent with the study by Rungapiromnan et al. (33) which demonstrated that secukinumab and ixekizumab did not significantly affect the risk of MACEs in adult patients with psoriasis in the short term, and there was no dose-dependent increase in the incidence of adverse events.

### 4.3 Limitation

The baseline characteristics of the included literature in this study were comparable, ensuring reliable results, high literature quality, and a large sample size. However, certain limitations should be acknowledged:

- Methodological Heterogeneity: The use of diverse follow-up treatment regimens introduced substantial methodological heterogeneity. Ethical considerations prevented the long-term use of a placebo, resulting in a relatively short trial period focused solely on MACEs during the double-blind phase, without further analysis.
- Age Representation: The predominantly middle-aged participants included in this study may not accurately represent the true incidence across all age groups. The incidence of such events tends to increase with age, and therefore, the finding may be limited in their generalizability to different age ranges.
- Cardiovascular Risk Factors: It’s essential to note that most patients had pre-existing cardiovascular risk factors. While myocardial infarction and stroke were identified as the most common cardiovascular events, the occurrence of these events may not be solely attributable to the administered drugs. The presence of these pre-existing risk factors introduces a potential confounding factor should be taken into consideration when interpreting the results.

### 4.4 Recommendations

Future research directions should encompass RCTs involve a broader spectrum of age groups among patients. This would help provide more comprehensive insights into the impact of IL-17A inhibitors on cardiovascular events across different age range. Furthermore, there is a need for more direct comparisons between different drugs to enhance the understanding of their relative efficacy in treating psoriasis and their effect on CV outcomes. Additionally, it would be valuable to conduct additional trials with consistent follow-up regimens, allowing for a more in-depth exploration of associated long-term and post-discontinuation safety concerns with IL-17A inhibitors. It is imperative that these studies are substantiated by an increased number of high-quality clinical trials to ensure the reliability and robustness of the findings.

## 5 Conclusion

A meta-analysis was undertaken to explore the impact of IL-17A inhibitors (secukinumab and ixekizumab) on serious cardiovascular adverse events in adult patients with psoriasis. To ensure the validity and reliability of the clinical evidence in this study, only randomized controlled trials were included, minimizing the potential sources of bias. This rigorous approach provides a solid foundation for assessing the short-term effects of IL-17A inhibitors on cardiovascular disease in individuals with psoriasis. Furthermore, the majority of the incorporated studies featured substantial sample sizes, enhancing the study’s utility as a valuable guide for informed decision-making, particularly when addressing comorbidities associated with psoriasis.

## Data availability statement

The original contributions presented in the study are included in the article/Supplementary material, further inquiries can be directed to the corresponding author.

## Author contributions

YZ: Writing – original draft, Investigation, Resources. ZY: Data curation, Writing – review & editing, Formal analysis. JG: Writing – review & editing, Methodology, Conceptualization. DS: Conceptualization, Writing – review & editing.
