# Supplementary material for: Effects of secukinumab and ixekizumab on major adverse cardiovascular events in patients with psoriasis: a meta-analysis of randomized controlled trials
Source: Front Med (Lausanne). 2024 Mar 6;11:1353893. doi: 10.3389/fmed.2024.1353893 (PMC10951107; doi:10.3389/fmed.2024.1353893)
Supplement: Supplementary file 1 [file Table_1.docx]

Supplementary Material

Effects of secukinumab and ixekizumab on major adverse cardiovascular events in patients with psoriasis: a meta-analysis of randomized controlled trials

**Yonghong Zhang1, Zhiya Yang2, Jinyan Gong1, Dongmei Shi2,3***

*** Correspondence:** Dongmei Shi, Email: shidongmei28@163.com.

# Supplementary Table 1

| First Author |  |  |  | Age, y | Duration of |
| --- | --- | --- | --- | --- | --- |
| surname and Year | country | design | group |  | psoriasis, y |
| Gordon, K. B., 2016 | United States | RCT | UNCOVER-1 |  |  |
|  |  |  | ①Ixekizumab treatment group（Q4W） | 46±13 | 19±12 |
|  |  |  | ②Ixekizumab treatment group（Q2W） | 45±12 | 20±12 |
|  |  |  | ③Placebo control group | 46±13 | 20±12 |
|  |  |  | UNCOVER-2 |  |  |
|  |  |  | ①Ixekizumab treatment group（Q4W） | 45±14 | 19±13 |
|  |  |  | ②Ixekizumab treatment group（Q2W） | 45±13 | 18±12 |
|  |  |  | ③Placebo control group | 45±12 | 19±13 |
|  |  |  | UNCOVER-3 |  |  |
|  |  |  | ①Ixekizumab treatment group（Q4W） | 46±13 | 18±12 |
|  |  |  | ②Ixekizumab treatment group（Q2W） | 46±13 | 18±12 |
|  |  |  | ③Placebo control group | 46±12 | 18±13 |
| Griffiths, C. E. M., 2015 | United Kingdom | RCT | UNCOVER-2 |  |  |
|  |  |  | ①Ixekizumab treatment group（Q4W） | 45±14 | 19±13 |
|  |  |  | ②Ixekizumab treatment group（Q2W） | 45±13 | 18±12 |
|  |  |  | ③Placebo control group | 45±12 | 19±13 |
|  |  |  | UNCOVER-3 |  |  |
|  |  |  | ①Ixekizumab treatment group（Q4W） | 46±13 | 18±12 |
|  |  |  | ②Ixekizumab treatment group（Q2W） | 46±13 | 18±12 |
|  |  |  | ③Placebo control group | 46±12 | 18±13 |
| Leonardi, C., 2012 | United States | RCT | ①Ixekizumab 150 mg treatment group | 46±13 | 15±10 |
|  |  |  | ②Ixekizumab 75 mg treatment group | 46±13 | 13±10 |
|  |  |  | ③Ixekizumab 25 mg treatment group | 46±15 | 18±11 |
|  |  |  | ④Ixekizumab 10 mg treatment group | 48±11 | 21±12 |
|  |  |  | ⑤Placebo control group | 45±13 | 15±11 |

**Supplementary Table 1.** Characteristics of the included studies.
